# Modeling DGNSS Pseudo-Range Correction Messages by Utilizing Satellite Repeat Time

**DOI:** 10.3390/s17040834

**Published:** 2017-04-11

**Authors:** Dong-Hyo Sohn, Kwan-Dong Park, Hyunu Tae

**Affiliations:** 1 Department of Geoinformatic Engineering, Inha University, Incheon 22212, Korea; dhsohn5@gmail.com (D.H.S.); hyunwoo313@nate.com (H.T.); 2 Earthquake and Volcano Research Division, Earthquake and Volcano Center, Korea Meteorological Administration, Seoul 07062, Korea

**Keywords:** DGNSS, GNSS, GPS, BeiDou, pseudo-range correction

## Abstract

We developed and validated a pseudo-range correction (PRC) modeling system that can prevent degradation of positioning accuracy even in situations where one cannot obtain PRC messages for Differential Global Navigation Satellite System (DGNSS). A PRC modeling scheme was devised based on the repeat time of GNSS satellites and previously-collected PRC data. The difference between the modeled and real PRC values observed at the reference station showed a bias error of about ±1.0 m and a root mean square error (RMSE) less than 1.5 m. When we applied the predicted PRC to Differential Global Positioning System (DGPS) and Differential BeiDou (DBDS) positioning, horizontal RMSE values were at a level of 1.0 m, while vertical RMSE was in the range of 1.8–3.0 m. We found that modelled PRCs can provide positioning results similar to those based on real PRCs and can provide significant improvement over standalone positioning without PRCs.

## 1. Introduction

Global Navigation Satellite System (GNSS) users can select an optimal data processing technique according to the required positioning accuracy, data processing time, etc. Among many techniques, the Differential GNSS (DGNSS) technique using pseudo-range correction (PRC) has been widely used in a number of fields because it can improve positioning accuracy in real time using a low-cost receiver. Recently, DGNSS positioning algorithms are considered as a critical component of sensor fusion for autonomous vehicle navigation [1,2,3]. To improve positioning accuracies, quite many studies of coupled GNSS and multi-sensors integration [3,4] and reduction of the multipath error [1,5] have been published. GLONASS, BeiDou, and Galileo signals in addition to GPS are used in multi-GNSS positioning to improve accuracy, reliability, and availability of DGNSS positioning [6,7,8].

DGNSS is a code-based relative positioning technique that employs two or more receivers simultaneously tracking the same satellites. PRC values are generated at a reference station whose precise coordinates are known beforehand, and then transmitted in real time to the user’s receiver through a choice of transmission media. Positioning accuracy ranges from less than a meter up to a couple of meters, depending on the performance of the receiver, the baseline length between the base and rover sites, and the transmission rate for PRC [9,10]. Thus, DGNSS has been widely used in various fields, such as car navigation and location-based services as well as surveying, aviation, logistics, etc. It basically assumes that GNSS errors at the base station and the rover are highly correlated. However, range corrections to the satellite can be spatially de-correlated with separation of the user from the reference station, and temporally de-correlated with the latency of PRC [11,12].

The PRC information created at the reference station is provided via communications links, such as a radio beacon, Networked Transport of RTCM via Internet Protocol (NTRIP), Digital Multimedia Broadcasting (DMB), Radio Data System (RDS), FM Data Radio Channel (DARC), etc. DGNSS users obtain higher accuracy using PRC data continuously received through the transmission media. However, positioning accuracy can be significantly degraded if PRC data cannot be transmitted due to radio interference or loss of correction signals. The expected accuracy with DGNSS is typically 1–3 m, whereas it can degrade to as much as 10 m without PRC [13,14,15]. In this regard, continuous reception of PRC is essential to acquiring the expected DGNSS performance.

A number of studies have been conducted to model and generate PRC values, even in situations where corrections cannot be received. Two of the most notable schemes are based on neural networks and the Kalman filter. Neural networks can predict future data using an optimized modeling system by repeated training based on past values. Therefore, this system needs time for training in order to have an effective prediction time. Case studies using an autoregressive moving average [16,17] and recurrent neural networks [18,19] showed that the predicted PRC had a range error of about 1.0 m. The Kalman filter is a method to recursively update estimates of the system parameters by processing successive measurements. It uses a series of measurements observed over time, and produces estimates of unknown variables. Mosavi [18] used the Kalman filter method in Differential Global Positioning System (DGPS), and showed that RMS errors of predicted values were less than 0.4 m.

Even though a neural network algorithm and a Kalman filter showed a prediction error of less than ~1.0 m, those two methods require PRC observations collected right before modelling starts, and thus work only for a relatively short period of time after data loss. For this reason, we devised an algorithm with which one can obtain PRC estimates at any desired epoch through a simple mathematical formula. In our implementation, a DGNSS server produces modelling parameters based on repeating patterns of PRCs. The user needs to access those parameters only once and can produce reliable PRC values when PRCs are not available.

In this paper, we present new algorithms to generate real-time PRC predictions for GPS and the BeiDou navigation satellite system (BDS), and we conducted validation tests. First, we briefly introduce the repeating patterns of PRC, and we show how to obtain their repeat times. For repeat-time estimation, rapid or ultra-rapid GNSS orbits in Standard Product 3 (SP3) format are used. PRC parameters per satellite are calculated using past PRC data and the orbital period of the satellite. Using these parameters, we predict PRC values for a single day, and we then analyze their accuracy by comparing them with real values generated at the reference station. In addition, DGNSS positioning accuracy was evaluated using predicted PRC.

## 2. Pseudo-Range Correction

**Figure 1:**
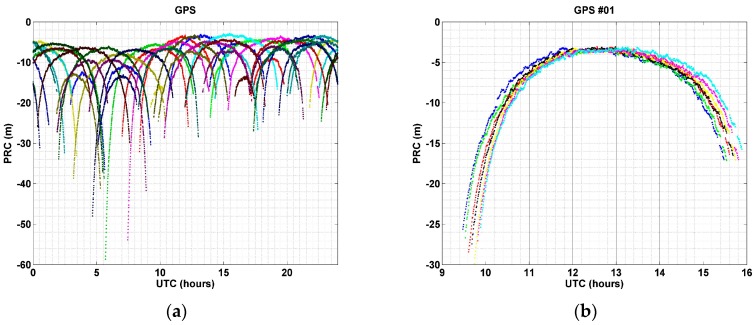
PRC observed at a base station in Incheon, Korea: (**a**) PRC of all GPS satellites for 24 h (23 April 2016). Each satellite is displayed with a different color; (**b**) PRC of GPS PRN 1 for a week (1–7 April 2016). Each color line is a different day.

PRC data contain a variety of types of errors related to range errors between the receiver and the satellite. Among the error sources, ionospheric and tropospheric delays are the two most significant ones. Since they are caused by atmospheric composition, the amount of a ranging error varies depending on the degree of solar activity and the length of the signal transmission. Therefore, PRC values also vary according to observation time and elevation angle of the satellite. In addition, PRC has a feature that is repeated periodically, because GNSS satellites orbit at a regular period. Figure 1 shows these characteristics.

Figure 1a shows PRC values of all visible GPS satellites for one day (23 April 2016) from the reference station at Inha University, South Korea (37.449° N, 126.656° E). As shown in Figure 1a, the relative maximum or minimum PRC is different according to the observation time. The reason for this difference is ionospheric error, which is strongly affected by the Sun. During 4:00–9:00 Universal Coordinated Time (UTC), which corresponds to 13:00–18:00 local time (LT) in South Korea, magnitudes of PRC are the largest. In particular, the PRC of some satellites shows PRCs larger than 50 m from 6:00 to 8:00 UTC (15:00–17:00 LT). On the other hand, it is less than 20 m, and some go down to 3 m during the period 12:00–21:00 UTC (21:00–06:00 LT). Thus, observation time is one of the important factors for determining PRC values.

Figure 1b shows PRC values of the GPS Pseudo Random Noise (PRN) No. 1 satellite for seven consecutive days (1–7 April 2016), i.e., for a week. As shown in Figure 1b, the shape of long-term PRC data displays the upside down U patterns repeatedly with a certain time difference. PRCs increase higher than 25 m at 9 to 10 UTC, when the satellite is observed for the first time, but become smaller as the elevation angle of the satellite becomes higher. PRC values were in the range of 3–4 m at 12 to 13 UTC, when the elevation angle was the highest. After that, however, it becomes larger again as the elevation angle decreased. This phenomenon was repeated every day with similar shapes for a week. As such, PRC is different according to the elevation angle of the satellite, and revealed a repetitive shape. Therefore, the orbital period of a satellite is one of the most important factors that determine the repeatability and periodicity of PRC.

## 3. GNSS Orbit Repeat Period

GNSS satellites are designed to orbit Earth for a regular period. However, the repeat period of a GNSS satellite observed from the ground does not match the orbital period owing to the Earth’s rotation effect. Moreover, even satellites in the same navigation satellite system have slightly different orbital periods owing to the effects of gravity variability and other perturbations. Therefore, a GNSS’s orbital period needs to be calculated for each satellite, considering the location of satellites observed from the ground, in order to perform modeling based on past PRC data. Here, we obtain a repeat time for when the satellite has the same aspect [20,21].

In general, GNSS users can choose almanac, broadcast navigation message, or precise orbits in order to calculate satellite positions and predict their orbits. Almanac consists of Keplerian elements, which can propagate GNSS satellite orbits in Earth-centered Earth-fixed (ECEF) coordinates. The broadcast ephemeris, on the other hand, provides a more accurate description of the satellite trajectory in real time. These data are essentially similar to almanac, but provide a better representation of the GNSS orbit by including secular and periodic perturbations [22]. In the case of International GNSS Service (IGS) orbits whose accuracy is higher than the previous two kinds, coordinates of satellites are directly recorded as X, Y, and Z values so that orbit propagation based on Keplerian elements is not necessary. Instead, a proper interpolation scheme should be adopted to compute the satellite position at a desired epoch.

We used precise SP3 orbit files provided by the IGS. SP3 files are classified as ultra-rapid, rapid, and final products according to accuracy and update period. In the present study, rapid products were used, where accuracy is similar to that of final products and updated once a day [23].

### 3.1. GPS

A GPS satellite has an orbital period of 11 h 58 min 2 s, and thus, orbits Earth twice a day. The length of a sidereal day is 23 h 56 min 4 s, which is shorter than a solar day by 3 min 56 s. Due to this time difference, satellites should be observed in the same direction at a slightly earlier time on the next day. Even though GPS constellations were designed with a regular orbit period, an exact repeat period of each satellite is slightly different due to a number of factors. In order to calculate a repeat time for each satellite, a time lag with which the cross-correlation becomes the highest should be determined in the X, Y, and Z directions using three-day SP3 files prior to the desired date, and the results are averaged. The reason for selecting a three-day dataset is to minimize errors by securing as much data as possible and to shorten processing at the same time.

A repeat time for GPS satellites calculated using cross-correlation was within the range of 220–248 s. These values differed slightly, according to the date and satellite. On average, GPS repeat time was found to be around 240 s. This value is similar to those published by Park et al. [24] and Agnew and Larson [21], who used repeat time in analyzing and removing multipath error.

### 3.2. BDS

The BDS space segment consists of satellites deployed in three different types of orbit [25]. The three orbit types are geostationary Earth orbit (GEO), inclined geosynchronous satellite orbit (IGSO), and medium-Earth orbit (MEO). The GEO satellites operate at an altitude of 35,786 km over the equator, and are positioned at 58.75° E, 80° E, 110.5° E, 144° E, and 160° E. Thus, three to five BDS satellites can always be observed from many Asian countries, including Korea. The orbit plane of IGSO satellites is inclined at 55° to the equatorial plane, although it is located at the same altitude as GEO satellites. The ascending nodes of IGSO satellites are located at 95° E and 118° E. MEO satellites are deployed in 21,528 km altitude orbits inclined at 55° to the equator. MEO satellites have an orbit period of approximately 12 h and 53 min, so the satellite ground tracks repeat every seven days. During the period, MEO satellites make 13 revolutions.

**Figure 2:**
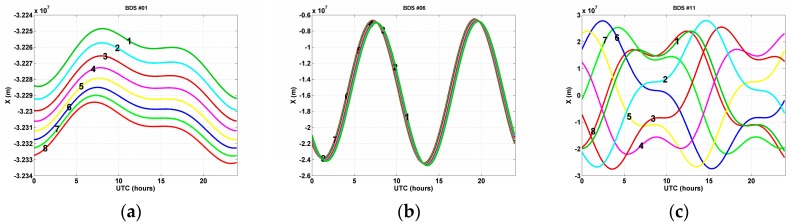
Eight-day time series of the X coordinates of satellite positions according to the BeiDou orbit type: (**a**) PRN-C01 GEO satellite; (**b**) PRN-C06 IGSO satellite; (**c**) PRN-C11 MEO satellite. The number inside each plot refers to the sequential order of dates of observations. Each color line is a different day.

The repeat time of a BDS satellite is a variable quantity depending on altitude and orbit type. Figure 2 shows X components of the satellite position by orbit type during an eight-day period. As can be seen from Figure 2a,b, GEO and IGSO satellites go through exactly the same tracks with a slight time lag. However, MEO satellite PRN #11 in Figure 2c shows a different trajectory from the other two. After seven days, the eighth orbit is similar to that of the first day.

Repeat times of GEO and IGSO satellites were calculated based on three-day data prior to the desired date. On the other hand, a repeat time for the MEO satellite was obtained by using data from 7 and 14 days previously. The reason for using only two-day data was that the MEO satellite has a longer repeat period than the other types, thereby making atmospheric conditions significantly different. From computation of cross-correlations, we found that GEO and IGSO satellites have repeat times in the range of 217–252 s, whereas MEO satellites have a repeat time of around 25 min (1490–1539 s) after shifting seven days.

## 4. PRC Modeling

### 4.1. PRC Data Collection

The PRC data used in the present study were collected from the Javad Alpha GNSS receiver and Javad GrAnt antenna permanently installed at Inha University, South Korea. PRC modelling experiments were conducted on 23 April 2016. For the GPS, three-day PRC data prior to the experiment date were used. However for BDS, three-day data as well as data collected 7 and 14 days earlier than 23 April were utilized.

### 4.2. PRC Modeling

**Figure 3:**
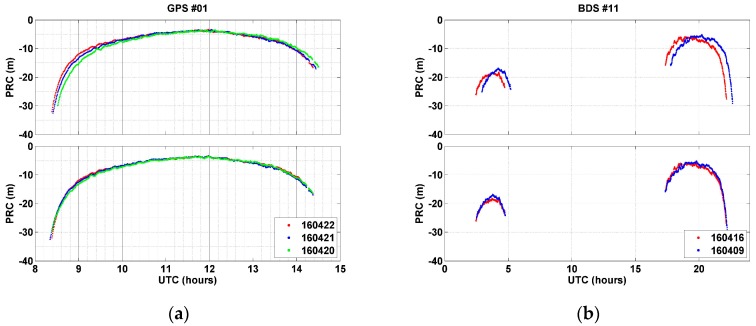
Alignment of PRCs before (top) and after (bottom) application of repeat times: (**a**) PRC of GPS PRN 1 for three days prior to 23 April 2016; (**b**) PRC of BeiDou PRN-C11 (MEO) from 7 and 14 days before 23 April 2016.

The repeat time obtained for each satellite was applied in shifting the PRC data collected before the experiment date. Figure 3 compares the time series of PRCs before (top) and after (bottom) aligning them based on the computed repeat time. Prior to applying a repeat time, although the PRC patterns were similar, a time lag was observed. On the other hand, after alignment, time lags almost completely disappeared, and overlapping and exactly matched shapes showed up. The PRC values that were shifted after applying repeat times were used to calculate modeling coefficients by means of polynomial curve fitting. The mathematical equations used for modeling can be found in [26]. In order to find the optimal degree of the fitting polynomial function, degrees from the 4th through the 12th were tried. The effectiveness of the fitting function was evaluated via root mean square error (RMSE) of residuals and data processing time.

**Figure 4:**
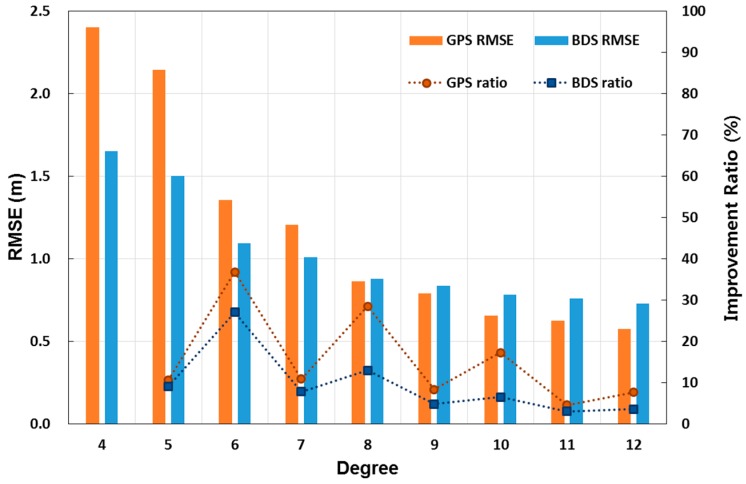
Mean RMSE values of all satellites after polynomial curve fitting to GPS and BeiDou. The left vertical bars refer to RMSE, and the symbols connected by dotted lines refer to improvement ratios in RMSE compared to those corresponding to the polynomial one degree lower. The improvement ratio was the highest when the sixth-degree polynomial was used.

The data processing times from curve fitting according to each polynomial degree were measured, and we found that the computation time slightly increases as the degree increases. However, no significant difference was observed, and the boundary that can help in choosing an optimal degree was not clear either. On the other hand, RMSE values after fitting showed a clear difference, depending on the degree of the polynomials. Figure 4 shows mean RMSE values averaged for all the satellites modelled. In this figure, the vertical bar indicates RMSE in each of the satellite navigation systems. The RMSE of the two systems became gradually smaller with the increasing degrees. In particular, GPS RMSE was larger than BDS RMSE at less than the eighth degree, whereas GPS RMSE values were smaller above the eighth degree. As shown in the figure, PRC fitting of the GPS was better than BDS as the degree became higher. A dotted line in Figure 4 denotes an improvement ratio in RMSE, compared to the previous degree. The largest improvement ratio was found at the sixth order in both systems. When we applied even number-degree polynomial functions, the improvement ratio was better than in cases with an odd number. This was because PRC values of all satellites, except for the BDS GEO satellite, have the upside down U shapes, resulting in better matches at even-numbered degrees. In the present study, we selected the sixth degree as optimal, considering the improvement ratio as well as the applicability to both systems.

### 4.3. Accuracy Analysis

**Figure 5:**
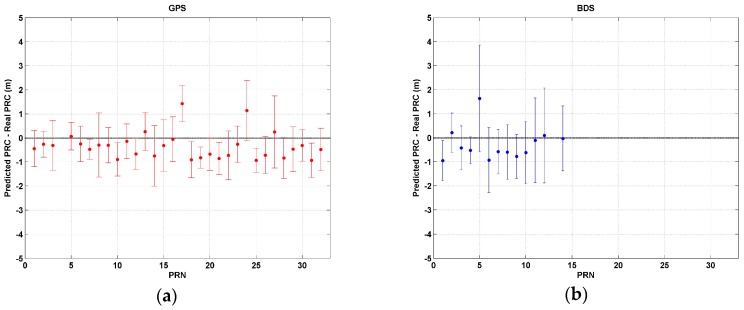
Mean value (bullet point) and standard deviation (error bar) of differences between real and predicted PRC values from (**a**) GPS satellites and (**b**) BeiDou satellites.

PRC modeling was performed by applying sixth-degree polynomial curve fitting, and satellite-dependent coefficients were derived. The accuracy analysis for PRC modeling was performed by comparing predicted PRC values at all epochs for one day during the experiment date with the corresponding real PRC data obtained from the reference station. Estimates were obtained using the coefficients derived for each satellite and the mathematical equations described in [26]. Figure 5 shows means and standard deviations of differences between real and modeled PRC values for every GPS and BDS satellite in our analysis. Except for a couple of cases, every GPS satellite has a bias error less than ±1.0 m and a standard deviation within 1.5 m. Overall, the average bias and standard deviation of GPS modeling is −0.4 m and 1.0 m, respectively (Figure 5a).

BDS also shows a similar level of accuracy, such as a −0.3 m bias and a 1.4 m standard deviation, on average. However, the PRN-C05 GEO satellite, which was always located at an elevation angle of around 9.4°, showed a 1.7 m bias and a 2.7 m standard deviation. It is interesting to find that standard deviations of MEO satellites from PRN C11 through PRN C14 are relatively high, at 1.7 m (Figure 5b). Those MEO satellites make the same orbit in seven days, so changing tropospheric and ionospheric conditions must have impacted modeling accuracy. The day of modeling, 23 April 2016, was a quiet day with the Kp-indices of global geomagnetic activity was smaller than 3.0 except for 21–24 UTC (Kp = 4.7). On 2 April, when the Kp-index was as high as 5.0, PRC residuals for individual satellite are within the range of ±1.0 m on average and the standard deviation was less than 1.5 m. Thus, no significant differences were observed for two dates with different levels of ionospheric activity. However, in order to evaluate the performance of modelling in general, further experiments are needed for the extremely high Kp-index.

**Figure 6:**
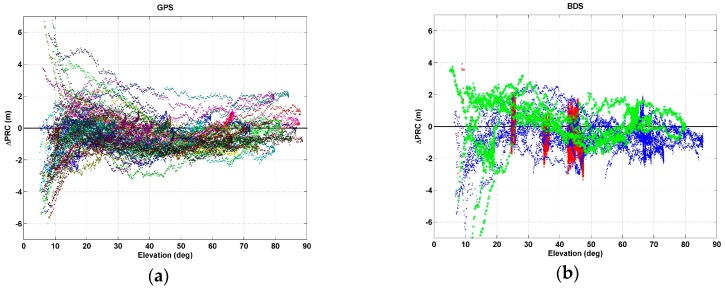

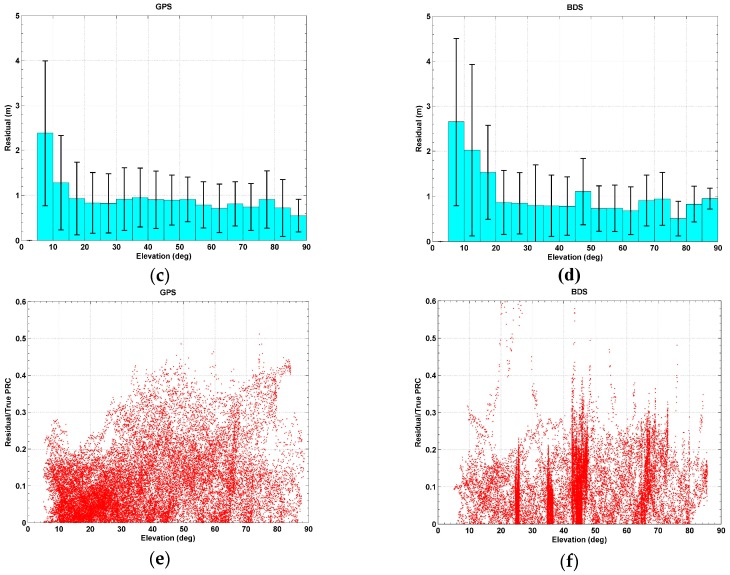
Differences between actual and predicted PRC values according to the elevation angle of (**a**) GPS satellites and (**b**) BeiDou satellites; (**c**,**d**) are the mean (bar) and standard deviation (error bar) of residuals for each elevation angle range; (**e**,**f**) show the relative error ratios.

After modeling with sixth-order polynomial curve fitting, differences between observed and predicted PRC values were found to be relatively large in the low elevation-angle range. Figure 6 shows discrepancies between observed and predicted values as functions of the elevation angle. GPS residuals are shown in Figure 6a, and the differences usually locate within ±2.0 m when the elevation angle is 20° or larger. However, some satellites showed deviations larger than ±4.0 m when elevation angles were less than 20°. Figure 6b depicts PRC differences for BDS according to orbit type. The red ×, blue •, and green * in the figure indicate GEO, IGSO, and MEO, respectively. Overall, patterns of residuals are similar to those of the GPS. However, PRC values for GEO were different depending on the date and time, even though GEO satellites had a nearly constant elevation angle, depending on the location along the equator. As can be seen from Figure 6b, the vertical distribution of difference appears at a nearly constant elevation angle. Meanwhile, IGSO and MEO satellites showed differences within ±2.0 m, but some satellites deviated more than ±4.0 m in the region where elevation angle is less than 20°.

Figure 6c,d shows the mean (bar) and the standard deviation (error bar) of the absolute value of the residuals at 5° intervals of elevation angle. Statistically, the residuals were less than 2.0 m all segments except for GPS at less than 15° and BDS at less than 20°. However, in Figure 6e,f, which show the ratio of the residuals to the true values, a relatively large ratios are observed in the region of higher elevation angles. That is to say, the absolute magnitude of the error decreases as the elevation angle increases, but the relative error is larger at medium and high elevation angles. The reason for the high absolute value of the PRC error at low elevation angle is that as the elevation angle of the satellite decreases, the atmospheric path length of the signal becomes longer and the time rate of change of the range gets larger. According to Brunner and Gu [27], the signal delays vary from about 55 m to 300 m at the elevation angle of 15 depending on the signal frequency and the ionospheric activity.

### 4.4. Limitation of PRC Modeling

The PRC modeling introduced in this study is an estimation method using satellite repeat time and past data. This method has an advantage that the user can generate PRC corrections using the modeling parameters even in the case where the user does not receive the correction for a long time. However, there is a limitation in that modeling does not reflect the characteristics of an ever-changing atmosphere very well.

Generally, the amount of signal delay due to the troposphere is about 2.3 m in the standard atmospheric condition [28]. Approximately 90% of the tropospheric delay is the dry or hydrostatic delay calculated as a function of surface pressure while the remaining 10% is the wet delay due to water vapor. Since the air pressure does not change very much day to day the dominant part of the tropospheric delay should have a very nice repeating pattern during several days.

The ionospheric delay can change significantly for a short period of time due to the solar explosion, geomagnetic storms, and so on. In those cases, a modeling scheme based on the past data may not work as nicely as expected. In an environment where no PRC information can be received at all, however, it should be possible to obtain better positioning results by using estimated PRC because at least the tropospheric delay can be compensated. Also, a more reliable DGNSS service scenario can be devised by monitoring the ionospheric disturbance and issue a warning message.

## 5. DGNSS Positioning

In this section, we assess the PRC modeling performance through DGNSS positioning using three methods, and their accuracies are compared. The three methods are (1) point positioning without PRC; (2) DGNSS with modelled PRC; and (3) DGNSS with real PRC generated at the base station. The mathematical model used for positioning is as follows [28]:

(1) $$R_{r}^{s}\left(t\right)=\varrho_{r}^{s}\left(t\right)+\Delta \varrho_{r}^{s}\left(t\right)+\Delta \varrho^{s}\left(t\right)+\Delta \varrho_{r}\left(t\right)$$

(2) $$PRC^{s}\left(t\right)=-\Delta \varrho_{r}^{s}\left(t\right)-\Delta \varrho^{s}\left(t\right)-\Delta \varrho_{r}\left(t\right)$$

where, $R_{r}^{s}\left(t\right)$ is the code range at receiver *r* to satellite *s* measured at epoch *t*, $\varrho_{r}^{s}\left(t\right)$ is the geometric range between receiver and satellite, $\Delta \varrho_{r}^{s}\left(t\right)$ is the satellite-receiver-dependent range bias (e.g., atmospheric refraction effects), $\Delta \varrho^{s}\left(t\right)$ is the purely satellite-dependent range bias (e.g., satellite clock error, orbit error), $\Delta \varrho_{r}\left(t\right)$ is the purely receiver dependent range bias (e.g., receiver clock error, multipath), and $PRC^{s}\left(t\right)$ is the pseudorange correction for satellite *s* at epoch *t*. DGNSS position solutions were obtained by least-squares estimation at every epoch and no weights were given to the measurement.

Positioning was conducted at the IGS station DAEJ located in Daejeon, South Korea, which was considered a rover and located approximately 133 km away in a southeasterly direction from the reference station at Inha University. The rover site is equipped with a Trimble NetR9 receiver and a TRM59800.00 antenna. The data sampling interval was 30 s.

### 5.1. DGPS Positioning

**Figure 7:**
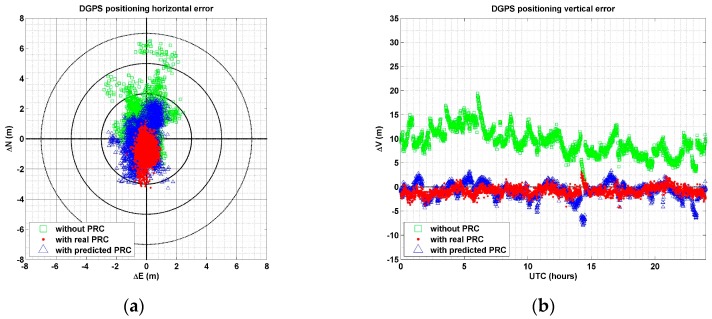
DGPS positioning: (**a**) horizontal and (**b**) vertical error without PRC (□), with a predicted PRC (△), and with the real PRC (•).

Figure 7 shows (a) horizontal (north-east direction) errors, and (b) vertical errors from three different positioning schemes. In this figure, the green square refers to standalone positioning errors without PRC, whereas the blue triangle and red dot refer to errors in DGPS positioning with predicted and observed PRCs, respectively. The concentric circles refer to horizontal error magnitudes of 1.0, 3.0, 5.0, and 7.0 m.

As shown in Figure 7, the most accurate result was achieved when real PRC data were used. Horizontal RMSE were 1.80 m for positioning without PRC, 1.39 m for positioning with a predicted PRC, and 1.00 m for positioning with the actual PRC. Although RMSE of DGPS positioning with predicted PRC was 0.39 m larger than with observed values, it improved by 0.41 m, compared to positioning without PRC. In terms of precision or standard deviation, the best case was again when the real PRC was used. Although most positioning errors were distributed within 5.0 m when PRC was not used, errors larger than 6.0 m showed up. In contrast, all errors (except some) were within 3.0 m when predicted PRCs were used, and most errors were distributed within a range of 2.0 m, although a bias of about 0.7 m occurred in the southerly direction.

The vertical errors showed that RMSE without PRC was 9.70 m. However, when predicted and observed PRCs were used, vertical errors are all below 2 m. Vertical error using predicted PRC was 0.57 m larger than with observed values. However, it improved to more than four times that of positioning without PRC. Since the vertical component in the coordinates was closely related to tropospheric delay, vertical errors were significantly reduced due to the effect of tropospheric error correction by PRC [13,28].

### 5.2. DBDS Positioning

**Figure 8:**
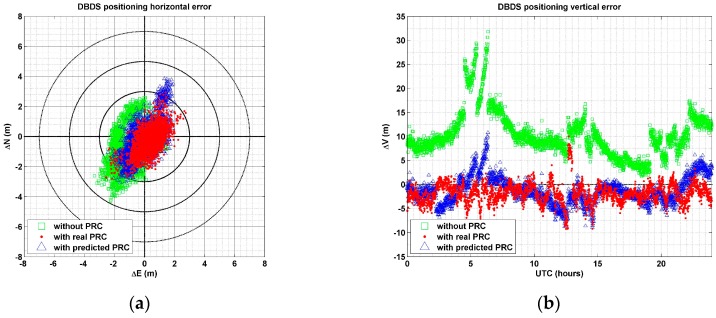
DBDS positioning: (**a**) horizontal and (**b**) vertical error without PRC (□), with a predicted PRC (△), and with the real PRC (•).

Coordinate accuracy processed with the three methods was compared based on the same BDS data used in the evaluation of DGPS positioning accuracy. Figure 8 presents a comparison of the three cases. The symbols used in Figure 8 are the same as those used in Figure 7.

In the DBDS positioning result, using the real PRC had the most accurate results. RMSE accuracy in the DBDS system with real PRC was 1.14 m, but accuracy with predicted PRC and without PRC were 1.40 m and 2.01 m, respectively. Although RMSE of DBDS positioning calculated with the predicted values was 0.26 m larger than from using observed values, it improved by 0.61 m, compared to standalone positioning without PRC. As shown in Figure 8a, most results using observed and predicted PRC showed errors within 3.0 m, whereas results without PRC had errors of up to 5.0 m. Positioning accuracy using the predicted values is lower than with observed values, but the degradation is not significant at all.

For the vertical direction, RMSE was 11.46 m when PRC was not used, but 3.05 m and 2.80 m when using predicted and observed values, respectively. The positioning result using predicted values was 0.25 m larger than from using observed values, but improved by 8.41 m from the standalone case without PRC. From the experiment, the effectiveness of PRC modeling was also verified through DBDS results. In particular, accuracy of the vertical component improved by about 70% compared to positioning results without PRC.

## 6. Conclusions

We have presented a new PRC modeling scheme that can generate correction information when users cannot receive PRC data. Predicted PRC through our modeling showed a bias error of less than ±1.0 m and an RMSE within 1.5 m, compared to actual PRC values from the reference station. Positioning accuracy using predicted PRC showed that horizontal RMSE for both DGPS and DBDS were 1.0 m, with vertical RMSE of 1.8 m and 3.0 m, indicating similar results from positioning with real PRC. From the above results, it was verified that modeling PRC values can be effectively used to maintain performance continuity in a DGNSS system, even in situations where correction data cannot be received. In particular, vertical accuracy was improved significantly with the prediction system.
